# Public Hygiène; or, Memoirs on the Most Important Questions of Hygiène Applied to Professions and to Works of Public Utility

**Published:** 1838-04

**Authors:** 


### Art. IX.

*Hygiène Publique; ou, Mémoires sur les Questions les plus importantes de la Hygiène appliquée aux Professions et aux Travaux d'Utilité publique.* Par A. J. B. Parent-Duchatelet, &c. &c. *Accompagné de* 48 *Planches. Précédé d'une Notice historique sur la Vie et les Ouvrages de l'Auteur, par* Fr. Leuret. Deux Tomes.—*Paris*, 1836. 8vo. pp. 552 et 708.

*Public Hygiène; or, Memoirs on the most important Questions of Hygiène applied to Professions and to Works of Public Utility.* By A. J. B. Parent-Duchatelet, &c. &c. *With* 48 *Plates. Preceded by an historical Notice of the Life and Works of the Author, by* Fr. Leuret. Two Volumes.

This large work is a monument of the extraordinary industry of its author in a department of observation which, although of immeasurable importance to the community, has never presented a very inviting aspect in this country, where it has, indeed, attracted very little attention. What has been called Political or State Medicine, but is now more commonly denominated Public Hygiène, although it relates to no less important a matter than the preservation of the health of the whole population, in all varieties of residence and occupation, is scarcely recognized as a study. Those who labour in unwholesome manufactures die in rapid succession, and no man cares, for workmen are never wanting. Neighbours quarrel about the site and erection of gas-works, and of tall chimneys, and juries are puzzled by the contradictions of medical witnesses, and property is depreciated or not as the accident of the verdict may determine. Barracks or camps are fixed in deadly spots, and the military hospital is filled; but great expense has been incurred, and the evil must be endured. Bad food and drink are retailed in great quantities, with perfect impunity except to the purchasers. Epidemics appear and disappear, here fatal, there harmless, and the lessons they teach are not collected for public benefit; and crowded and unventilated courts spring up in and around every street. The laws of quarantine, considered useless and absurd by some, are regarded as vital safeguards of our shores by others. Of several towns, the citizens are in the utmost state of habitual uncertainty respecting the goodness or injuriousness of the water used for daily drink. These, and numerous other public inconveniencies unnecessary to mention, are felt from year to year, and little or nothing is done to promote the kind of knowledge which might lead to their removal.

On the greater number of these points it may appear ungracious to say, what is nevertheless strictly true, that the opinions of medical witnesses are little to be relied upon, and that their general authority is unworthy of confidence; in proof of which it is only necessary to refer to the question of the salubrity of the Thames water, so strongly agitated ten years ago, and to the more recent question of the safety of riding through rail-road tunnels. Strong assertions, resting on unstable doctrines, may there be found in abundance; but of careful observation and wise experience there are few traces. Many of the witnesses seem to have been actuated by a love of almost theatrical display, and to have delighted in giving more force to popular prejudices and apprehensions; but teachers of valuable truths, important to the general health of the people, we find none. The evidence given during the enquiry made into the effects of certain parts of the factory system, although containing more information, presents equally glaring and disgraceful contradictions. Medical men, and the public in general, seem to come to the examination of these questions quite unprepared. Passion and various local influences, and many prejudices, mislead them; and they arrive at and announce no general principles that can be depended upon. Nothing can be so positive, and apparently so satisfactory, as the assertions made on either side, except the assertions made on the other.

Much of this would, we think, be obviated by making public hygiène a subject of more public interest; and no subject can deserve it more. "I have travelled," says M. Fodéré, one of the first among the French to shew the value of this study; "I have lived in different countries; I have reflected on the condition of man in the different circumstances of life; I have seen that it is in the power of governments to do them infinitely more good than all the books of medicine put together."—"There is a great department in the science of government," says Dr. Kilgour, who quotes this observation; "the application of legislation to relieve the physical necessities, extend the physical comforts, and improve the physical condition of the human race: in short, to make the public healthy, vigorous, and virtuous, in so far as moral perfection depends upon, or is connected with, the healthy condition of the body." Dr. Hawkins, in his writings on Statistics, has amply shewn the connexion existing between misery and disease; and that, wherever want and misery abound, there sickness, and miscarriages, and early death, and every indication of a degenerated people, are to be met with; but, whatever progress these principles may have made in the minds of intelligent people, an inspection of any of our towns will shew how much they are lost sight of. Large portions of the community of our highly civilized country are continually exposed, and perhaps needlessly, to causes which inevitably tend to make life wretched, and to shorten, first its efficacy, and then its duration.

The city of Paris has for nearly forty years enjoyed the advantage of a council of health (*conseil de salubrité;*) which at first consisted of only four members, whose duty it was to examine adulterated liquors, unwholesome workshops and manufactories, and the epidemic diseases of animals: they were subsequently charged with the visiting of prisons and the direction of public charities. It was afterwards found necessary to increase the number of its members to seven, and to enlarge the sphere of their labours. The treatment of epidemics was confided to them; and they were appointed to inspect the markets, rivers, cemeteries, slaughter-houses, dissecting-rooms, privies, sewers and collections of manure and filth, drains, pits, public baths, depôts of mineral waters, &c.; they were also to attend to medical statistics and the tables of mortality; to institute enquiries concerning the means of rendering workshops and public places wholesome: to take measures for preventing inundations, or repairing their effects; *to repress quackery;* to determine the best methods of warming and lighting, &c. &c. &c. These manifold offices led to the drawing up, in the course of fifteen years, of no fewer than

4,330 reports. The institution is said to have obtained the confidence of the public, and to have been usefully imitated in other countries, and in some of the departments.

M. Parent-Duchatelet well observes, that an ordinary medical education by no means forms a sufficient preparation for the duties of a member of a council of this kind. They demand a great deal of knowledge not to be acquired in the medical lecture-room or in the hospital, and, in particular, a knowledge of general physics seldom possessed by the medical practitioner, and a practical acquaintance with workshops and various other localities. In a country like England, where no such council exists, and where the practice of the medical profession is lucrative, it cannot be expected that many students will devote themselves to such complicated and difficult enquiries. But, as the welfare of the community imperiously demands that attention should be given to the subject, in all its details, we should be most glad to see the institution of a Board, the duties of which would be attended with a sufficient remuneration to induce medical men to pay more regard to hygienic studies. The number so doing would, we feel convinced, soon be considerable.

A great defect, however, exists in this country, in the omission, in any curriculum of study, of the subject of hygiène altogether. The chair of Hygiène in the Faculty of Medicine in Paris has been filled by distinguished men; among others, by Andral; and valuable works have been published on the subject. In America, also, hygiène receives much consideration, and professors are appointed to teach it in some of the colleges. We cannot but be of opinion that great advantage would accrue to the public from some encouragement being given to the study; and nothing would tend so much to the removal of the too-general want of information concerning public hygiène as making it one of the subjects to be included in the studies of every physician and surgeon. Its general principles might, at least, be correctly learned by every practitioner; although its ample details might only be embraced by a few, who would thereby qualify themselves for important public duties. We trust we are correctly informed that this is likely to be one of the benefits conferred upon the English community by the Senate of the Metropolitan University.

Our previous notice of M. Parent-Duchatelet's work, on Prostitution in the City of Paris, must have made our readers well acquainted with the nature of the patient and diligent enquiries to which he devoted his life. Of these, the two volumes before us are an extraordinary monument. They comprehend twenty-nine sections, of which the subjects are generally of much importance, and, although repulsive to many enquirers, to the elucidation of which the author applied himself with all the enthusiasm that might have been expected in more attractive investigations. Of grave and studious habits from his youth, and after a careful education, he took the degree of doctor of medicine in Paris, in 1814, and was for a few years engaged in practice. In 1821, he published, conjointly with M. Martinet, a treatise on Arachnitis. He appears, however, to have conceived some disgust for medicine, in consequence of its uncertainty as a science; and, from 1821 to 1836, he gave himself wholly up to the prosecution of hygienic enquiries; of which the results are the volumes which now occupy our attention and those reviewed in our Number for July, 1837. To whatever subject his enquiry was directed, he brought the same untiring industry and the same admirable method by which his researches concerning prostitution are characterized. He had even conceived a sort of fondness for situations and occupations which would be revolting to ordinary persons. In the midst of a fête at the Hôtel de Ville, burthened with a court dress, and embarrassed amidst a crowd of frivolous people, he whispered to a friend that he would rather go into a common sewer than into such a company. But in such a locality it was, even in a common sewer, that he had achieved some glory, or at least had essentially contributed to the public advantage. The Amelot sewer, one of the largest which flows beneath the Parisian capital, had, from long neglect, become totally obstructed. The consequences to those aboveground were hideous: cellars, houses, and streets were subjected to an obscene inundation. All cure of the evil seemed vain; many workmen were asphyxiated in the attempt, and the Amelot sewer became the dread of nightmen. It was thought that the remedying of the old sewer must be abandoned, and a new one constructed at an enormous expense. But the prefect of police appointed a commission, to undertake and direct, if possible, the renovation and purification of the sewer, with safety to the public health and also to the workmen. Of this commission Parent-Duchatelet was one; and under his direction this great and difficult work was undertaken, with so many precautions, and with so much care of the workmen employed, that, in the space of six months, the Amelot sewer and its branches were cleared of 2,150 cart-loads (*tombereaux*) of solid matter, and thrice the quantity of matters soft or semi-liquid; and, at the end of the task, the workmen were all in perfect health, and the whole expense incurred was only about 33,000 francs, (1,320*l.*)

Feeling all the importance of subjects of this kind, M. Parent remonstrates with the happy people enjoying themselves aboveground in Paris, concerning their small regard for those who toil in the rivers "dark and deep" below the soil,—those, without whose labours the air of the surface would soon become poisonous and the city a desert, and yet who are exposed to be carried away by the black and noisome currents which flow in that "sad Acheron," or suffocated by irrespirable gases, with no one to appreciate their useful labours or care for their fate. He reminds his readers that, among the ancients, the public salubrity was considered a matter of the first importance; that the Roman drains, among the wonders of antiquity, were under the protection of tutelary deities,—Sterquilinus, and Cloacina, and Mephitina; and that, among the Greeks, Epaminondas, and, among the Romans, Cicero and Agrippa, were superintendents of these essential structures. In the investigation of the drains of Paris, M. Parent visited them all, enquired of the workmen, compared their evidence, and spared neither time, nor money, nor labour, to attain the knowledge which he sought.

In all his investigations, disregarding mere conjectures and prepossessions, paying especial attention to facts, and applying to them, what in this instance was legitimately applied, the numerical method, he frequently arrived at conclusions opposed to all ancient prejudices. We pointed out some instances of this in noticing his work on Prostitution; and M. Leuret mentions another, which very well illustrates such results in relation to the actual influence of tobacco on the workmen employed in the different preparations undergone by this plant. The opinion of all authors who had spoken of its influence upon health was, that it was especially hurtful. Ramazzini, and after him Fourcroy, and later, Cadet-Gassicourt, Tourtelle, Percy, Patissier, and Merat had given the same testimony. Tobacco was accused of making the workmen emaciated, sallow, asthmatic, liable to colic, vomiting, intestinal hemorrhage, vertigo, headach, tremors, narcotism. Of all these crimes, hardly thought of in King James's "Counterblast," M. Parent, in conjunction with M. d'Arcet, acquitted tobacco, after the most diligent examination; although we do not think they have succeeded in freeing the plant from all suspicion of guilt. Numerous valuable facts, connected with this and other subjects, are recorded by M. Parent, in the "Annales d'Hygiène publique et de Médecine légale," of which useful publication he was one of the founders. Among these, the results of his investigation of the effects of water in which hemp has been steeped, may be mentioned as worthy of remark. The fountains of the town of Mans are supplied by water in which this process has been carried on; and a commission was appointed by the minister of the interior to examine into certain alleged inconveniences resulting from it; which inconveniences they pronounced to be imaginary. A similar opinion had previously been given by M. Marc, concerning the flax-ponds of Gatteville. To this enquiry, relative to a subject on which much difference of opinion was entertained, Parent zealously devoted himself for about two years; and he came to the conclusion that water in which hemp has been steeped is *not* hurtful to the health of those who drink it; nor more hurtful to fish than water which has been employed for other vegetable macerations; that it is not narcotic; and that air charged with emanations from hemp is not improper for respiration. His experiments in confirmation of these opinions were extended to himself, his wife, and his family, who, as well as others, drank flax-water, and slept in rooms with damp flax, &c., with perfect impunity.

The first of the thirty-nine sections of M. Parent's book consists of Considerations on the Council of Salubrity of Paris; the second relates to certain medical prejudices hostile to the salubrity of cities and manufactories; the third is occupied with the plan of hospitals for old and infirm persons; the fourth treats of methods of respiring deleterious gases with impunity; the fifth, sixth, and seventh relate to the healthiness of the city of Paris, as affected by the river Bièvre or Gobelins, and by the cloacæ or drains, including an account of the processes employed to cleanse the great Amelot drain. The eighth has for its subject the influence of feculent and marshy emanations on the public health; the ninth describes the Artesian pits employed for carrying off impure waters; the tenth contains an enquiry into the influence of dissecting rooms, and the means of purifying them. Every one of these sections is full of curious particulars; but we can only very briefly notice a few of them, or of the other memoirs; and we shall confine ourselves, as usual with us when noticing works comprehending so great a variety of topics, to those chiefly interesting to medical men, although there is a difficulty of selection in regard to the present work, since every section has some bearing on the means of promoting public health. From the general subjects, however, of the public health, M. Parent's observations, in his first section, are calculated to deter every practising physician; since, according to his views, it requires a variety of information which he cannot always devote himself to obtaining. To possess extensive medical literature, to be an excellent physician at the bedside, or an eloquent and able professor, do not alone form qualifications, he says, for one of the Council of Salubrity of Paris. A considerable acquaintance with the general physical character and constitution of the soil of the neighbourhood is necessary, and even of the geology of the neighbouring districts: above all, it is requisite to know the precise effects of professions and trades on the health of those engaged in them, and also of manufactories, &c. on the health of men collected in cities, and on animals; and this kind of knowledge cannot be acquired in the study, nor without exact notions concerning the arts and the greater number of particular processes of each trade. This kind of knowledge is not to be expected from mere physicians, but rather from those who have made public hygiène a special study, and from manufacturing chemists. Men so accomplished are, we fear, only to be expected in countries where councils or commissions are supported by the government, to pay especial attention to such subjects.

The section relative to the inconveniences of dissecting rooms, and the means of preventing them, is interesting to all who value the study of anatomy. It begins with an historical review of the Parisian school of practical anatomy; in which we are reminded that the first cultivators of this science in Paris had to encounter formidable obstacles, created by the ignorance and prejudice of the age, and were driven to pursue those investigations by stealth and in darkness, of which the results were discoveries that yet excite our admiration. In order to put a skeleton together, Vesalius passed whole nights in the charnel-houses of the cemetery of the Innocents, with much personal risk; and the same great anatomist laid the foundations of anatomical science, by resorting to the gibbets at Montfaucon, and disputing for the remains of criminals with vultures and animals of prey. This, we presume, refers to that period of the life of this most remarkable man when he studied under Sylvius at Paris, with so much ardour as to endanger his life; at a time when, no doubt, he was laying the foundations of the brilliant lectures on anatomy which afterwards attracted so many disciples to his theatre at Padua. But his life was not destined to be tranquil; and, having been to Palestine, for some unknown reason, either for the fulfilment of some vow, or, according to some authorities, to escape the indignation of the inquisition, excited by his having dissected a Spanish grandee, who proved not to be quite dead, he was, on his return from his pilgrimage, driven by a tempest upon the island of Zante, where he perished; so that, whether we regard his first stolen labours in Paris, when anatomy was considered unlawful and impious, or the supposed cause of his pilgrimage, and therefore of his death, he may be considered as having been an especial martyr to the science in connexion with which his name acquired immortality.

At a later period, at the beginning of the seventeenth century, when anatomy was protected in Paris by authority, the bodies of criminals alone were given for dissection. These were consigned to the physicians, between whom and the surgeons there was great hostility. But the surgeons wished also to teach anatomy, and, as bodies were scarce, they arrayed themselves against the physicians, and took subjects from them by force, until prevented by an act of the Parisian parliament. Still, for some time afterwards, whenever there was an execution in the Place de Grève, the students fought with swords and pistols for the body of the dead.

At the period when these strange scenes occurred, the common people often took the part of the students against the officers of justice; but, after the commencement of the eighteenth century, when the bodies of persons dying in the hospitals began occasionally to be dissected, the anatomical pupils lost the popular favour; and they have never regained it. The dissections, indeed, were yet conducted with many of the disadvantages attached to unlawful proceedings. Regular dissecting rooms there were none, and the students either pursued their researches in their own rooms, up many pairs of stairs, or in lofts, and granaries, and sheds, on portions of the human bodies furtively obtained. Gravediggers were in league with them, to baffle the churchwardens and despoil the graves; and no proceedings were taken against them, except when they were unlucky enough to be found out in some glaring act of resurrection. In M. Parent's researches into antiquarian collections of manure and filth, closed at the beginning of the reign of Louis XVIth, the frequent occurrence of human bones, and particularly of skulls, to which the saw had evidently been applied, furnished curious testimony to this part of dissecting-room history. It was not until the era of Desault, after the middle of the last century, that these clandestine proceedings became unnecessary. That celebrated surgeon established a large dissecting amphitheatre, in which there were generally not fewer than fifty or sixty bodies. Pelletan, Dubois, Lallemand, Boyer, Bichât, his contemporaries or the pupils of this more brilliant period, were led to imitate his exertions; hiring the upper stories of houses in situations little sought after, and establishing schools of practical anatomy. Bodies were supplied by depredations cleverly made on the churchyards, and were transported in hackney coaches: the remains were burnt; and, although the citizens in the neighbourhood were grievously incommoded by bad smells, the police were so careless of these proceedings that Professor Lallemand assured M. Parent they might at that time have killed as many people as they wanted, and have destroyed their remains in perfect security. It was not until after the commencement of the present century, in 1803, that the rooms of the dissectors were regularly placed under the superintendence of the police. Among the first regulations was one enjoining daily Guytonian fumigations, and another forbidding the dissection of those dying of contagious diseases. A later ordonnance enforced the fixing up of outside canvass blinds, as it was found that the windows commanding a view of what was going on in the dissecting rooms were for ever filled with women and girls, to the great interruption of domestic industry. The police found the dissectors unmanageable and contumacious. Nothing could exceed the filth, the neglect, and the indecency with which anatomical operations were carried on; and students, and dissecting-room porters, delighted to baffle and set at defiance the agents of justice, who, half afraid to enter these dens of every horror, complained that the living were scarcely more respected than the dead. It required the vigour of Napoleon, aided by the experience of Dupuytren, to restrain these disorders; and the subject of the centralization of the dissecting rooms was agitated from 1806 to 1810, when a plan for a new arrangement was proposed, comprehending a central institution on a very complete scale; but the expense, 884,534 francs, (above 35,000*l*.,) and probably other circumstances, prevented its adoption.

About this time the police discovered and put an end to a singular trade, which had long been carried on between the dissecting-room porters and sundry unsuspecting tradespeople and artisans of the good city of Paris; no other, in short, than the sale of melted human fat. This preparation was sometimes sold for the simple purpose of greasing the wheels of carts; but it was also bought by quack doctors, who sold it as a salve sovereign for aches and pains; and in much greater quantities by enamellers and workers in pearl; the first of whom purchased it for combustion when a great heat was required, thinking it no other than dog-grease or horse-grease. The trade was conducted on a great scale, and the depôt of the dissecting-room porters required a cart and two horses and three labourers for its removal. At the marriage of Napoleon with Maria-Louisa, the illumination-lamps of the Faculty of Medicine, and, less appropriately, of the palace of the Luxembourg, were supplied with a mixture of this notable product and tallow.

In order to check the numerous evils originating in private dissecting rooms, an ordonnance, dated in 1813, suppressed them all, and enjoined that dissections should be carried on in the amphitheatres of the Faculty of Medicine and the hospital of La Pitié alone: even the practice of operations on the dead body was forbidden, except in these authorized localities; but the rigorous conditions of this ordonnance were mitigated in 1814, at least in favour of the Bicêtre, to which M. Parent had been appointed physician. Much contention took place between the hospitals and the Faculty of Medicine, respecting the allotment of bodies. The regulation was, that the Faculty should have four-fifths, and the hospitals the remainder; but a more equitable arrangement was made in 1815, and has proved, we believe, generally satisfactory. Besides the amphitheatres of the Faculty and La Pitié, dissecting rooms for the use of the pupils of several of the hospitals are permitted to exist. The dissecting rooms of the Faculty are appropriated to those taking inscriptions, of whom, in the winter, there are about six hundred. The students from Great Britain and Ireland resort in great numbers to the dissecting rooms at La Pitié. For the rooms at the Faculty, a thousand bodies are required annually; and for La Pitié twelve or fourteen hundred.

With the intention of vindicating the practice of dissection from some of the charges brought against it as regards its effects on the health of the students, M. Parent enters into several curious particulars relative to the students of Paris, not unworthy, we think, of attention. He alludes to the well known fact that the majority of those who visit the French capital are soon attacked with diarrhœa and other inconveniences, in disproof of such affections being peculiar to the dissecting rooms. He is even disposed to deny the bad effects of the dissecting room emanations altogether. Although it is certain that either the air, or the diet, or the water, or the wine of Paris, produces violent temporary disorder of the bowels in many of the visitors soon after their arrival, it is no less certain that the same kind of disorder is produced in some susceptible individuals whenever they pass a few days in a dissecting room; and that the effects are only put an end to by absence, being again and again produced by a return to dissection. However, M. Parent affirms that not one in a hundred is affected with severe illness during the course of anatomical study, and his affirmation, we may rest assured, is made on calculations. The greater number of Parisian students he describes as poor; many of them living almost entirely on bread, sometimes with the addition of a little brandy; the clamours, indeed, of many of them who live a little better, when the dinner hour brings them together at the restaurateurs in the neighbourhood, indicate a degree of hunger as well as of ferocity, which, added to the suspicious viands brought forth to allay the excitement, make up a scene as repulsive as can well be imagined. But if these causes are not sufficient to disturb the health of these young followers of science, the coldness and dampness of the dissecting rooms, the long confinement to one position, and the mental anxiety, may well be supposed to do it. Some of the students also pass many hours of the day in the hospitals, the air of which, whatever it may do for the sick, has notoriously bad effects on those who are in tolerable health. The most frequent maladies among the students, M. Parent says, are cerebral and gastro-intestinal maladies, by which he means fevers with cerebral or intestinal complication. Phthisis is also common. But all these affections are met with in the schools and colleges, particularly in students whose curriculum is nearly completed. If, two or three months before the end of the session, several medical students are obliged to desist from their labours and go home, it is also found that of 130 young men at the seminary of St. Sulpice, twelve or fifteen are affected in the same manner every year. In addition to these observations, M. Parent quotes against the specific unhealthiness of dissecting rooms the unquestionable testimony of Boyer, Dumeril, Dupuytren, Serres, Lallemand, Dubois, Ribes, Roux, Beauchêne, Jadelot, Breschet, and Andral. Independently of this evidence of the innocuousness of human dissections, M. Rousseau, the chief preserver in the anatomical department of the Museum of Natural History, states that he has been occupied there for thirty-six years; that they often dissect large animals, lions, bears, camels, elephants, and that the bodies are kept a fortnight or three weeks in the hottest weather; that the dissectors work all the day, and stop not for putrefaction, though it distends, bloats, *greens*, and detaches the hair of the dead animals; and that, notwithstanding all this, and the penetration of bad smells into their houses, he and his colleagues, as well as his father for forty years before him, have enjoyed good health.

It is unnecessary to quote the numerous other instances given by M. Parent of uninterrupted health enjoyed by persons who may be said almost to have passed their lives within the dissecting-room atmosphere; for we believe little doubt is now entertained concerning such facts; and it is well known that several of the most distinguished pathological anatomists have lived to a good old age. The consideration of the results of wounds received in dissection did not form part of M. Parent's plan; such results not being to be ascribed to peculiar emanations. He enters fully into the subject of the means of keeping the dissecting rooms clean and wholesome, by ventilation, &c., although if we are to admit all the previous testimony to the fullest extent, such means would be more conducive to comfort than really important to health.

It was our intention to notice some other portions of M. Parent's work; but of the numerous details contained in the several memoirs which make up the two bulky volumes, few appear on examination to be unknown to medical men. Their collection attests the industry of the author, and the great attention paid to public health in the French capital. There is a short and interesting notice of the effects of the hasty burials of July, 1830, and of the subsequent and necessary exhumations. A memoir on the prevalence of ulcers of the legs among work-people contains some useful hints. So awake are the good people of Paris to hygienic precautions that even a poor beater of carpets cannot establish himself on a vacant spot of ground without becoming vexatiously involved. He selects what seems a happy locality; solicits and obtains leave from the commissary of police, and from the surveyor of the highways; sets up his lines, and commences active proceedings. Straightway the neighbours take the alarm; draw up a procès-verbal, and petition the council of salubrity. Five commissioners are appointed to look into the affair. The complaints of the excited neighbourhood resolve themselves into three heads; insalubrity, inconvenience, depreciation of surrounding property. Carpet-beating is suspended during the process of enquiry. First, as respects insalubrity; the dust of carpets, say the neighbours, consists of the fæcal dejections of animals, of moths and vermin; and these, borne on the wings of the wind, quickly destroy furniture, and even trees and plants; the same dust also absorbing in its flight the principles of many diseases, and thus causing inflammations of the eyes, cough, and pectoral irritation: it also drags with it woolly particles, which excite hæmoptysis, which brings on phthisis. In support of these charges they adduce some beaters of carpets themselves, found with some difficulty, but who, although receiving high wages, are pale, thin, and asthmatic. We must here observe, that M. Parent, not content with shewing that the dust of carpets is guiltless of many of these offences, insists on the perfect harmlessness of all the flocculent particles which thicken the atmosphere breathed by those who beat the hospital-mattresses, by beaters of the hair of cachemire and camel, of rabbit and of hare; of those employed in making hats; of those who drive in dusty roads, of those who live in the plaster-quarries, and of tailors in general. Herein we somewhat suspect the accuracy of M. Parent's enquiries. But the commissioners, rejecting the idea of the insalubrity of carpet dust, pronounce the noise of the trade insufferable, and hurtful to the value of surrounding land. The hammer produces no clatter so annoying as this. An ingenious beater of carpets lodged himself, years ago, beneath one of the arches of the Pont-Neuf, and laboured unceasingly from morning until night: but the whole neighbourhood cried out upon him: shopkeepers of the quai des Augustins were driven to desperation; inhabitants of the quai de la Monnaie could not hear themselves speak; and the police were obliged to unearth the poor carpet-beater for the peace of the parish. So it was also with the carpet-beater of the rue de Marbeuf, a more aristocratic quarter: he was pronounced a depreciator of property, his privilege was withdrawn, and he was compelled to decamp.

Without extending our notice of these Memoirs, we can safely recommend them for reference when any of our medical brethren are called upon to give an opinion concerning supposed nuisances of various kinds. It is possible that the subject may have been too minutely studied in Paris; but it has certainly not attracted sufficient attention in England.

---

